# Digital social entrepreneurship: the N-Helix response to stakeholders’ COVID-19 needs

**DOI:** 10.1007/s10961-021-09855-4

**Published:** 2021-03-30

**Authors:** María J. Ibáñez, Maribel Guerrero, Claudia Yáñez-Valdés, Sebastián Barros-Celume

**Affiliations:** 1 School of Business and Economics, Universidad del Desarrollo, Av. Plaza 680, Las Condes, Santiago Chile; 2 Northumbria Centre for Innovation, Regional Transformation, and Entrepreneurship (iNCITE). Business and Law Faculty, Newcastle Business School, Sutherland Building, 2 Ellison Pl, Newcastle upon Tyne, UK; 3 Centre for Innovation Research (CIRCLE), Lund University, Sölvegatan 16, Lund, Sweden

**Keywords:** Stakeholders theory, Digital social entrepreneurship, N-Helix collaboration, Knowledge transfer, Technology transfer, COVID-19 pandemic, F62 (Macroeconomic impacts), I18 (Public Health), I23 (Universities and Research Institutions), L26 (Entrepreneurship), L31 (Social Entrepreneurship), O31 (Innovation and Inventions), O33 (Technological change), O35 (Social Innovation)

## Abstract

This study explores the emergence of a new entrepreneurship phenomenon (digital social entrepreneurship) as a result of the collaboration among many agents (N-Helix), given the government’s limited capacity to respond to the stakeholders’ needs satisfaction related to an exogenous event (e.g., the COVID-19 pandemic). Our theory development is based on three ongoing academic debates related to (a) the unrepresentativeness of *the stakeholder theory* in entrepreneurship research; (b) the emergence of *digital social entrepreneurship* (DSE) as a bridge between stakeholders’ needs, socio-economic actors, and digital-social initiatives; and (c) the role of *N-Helix* collaborations to facilitate the emergence of global knowledge-intensive initiatives and the rapid adoptions of open innovations. Our results support our assumptions about the positive mediation effect of DSE in the relationship between N-Helix collaborations and stakeholders’ satisfaction. Notably, results show how pandemic has intensified these relationships and how DSE in N-Helix collaborations can generate social impacts globally. Some implications for policy-makers have emerged from our results that should be considered during/post-COVID-19 pandemic.

## Introduction

On January 1st, 2020, the Word Health Organization (WHO) announced a severe acute respiratory syndrome coronavirus 2 (SARS-CoV-2), which causes a new disease called coronavirus. Two months later, the WHO recognized coronavirus as a pandemic (the COVID-19) on March 11th, 2020. At this moment, we started to observe worldwide curfews, quarantines, and similar restrictions (i.e., stay-at-home orders, shelter-in-place orders, shutdowns, or lockdowns) related to the COVID-19 pandemic; consequently, from January 1st to July 31st, WHO has confirmed 17.064.064 cases of COVID-19 across 216 countries around the world, including 668.073 deaths (WHO, 2020b). According to the June 2020 World Bank Global Economic Prospects, the pandemic’s immediate impact was a 5.2 percent contraction in global GDP in 2020—the deepest global recession in decades. Despite governments’ efforts to counter the downturn with fiscal and monetary policy support (Frydman & Phelps, 2020), a deep recession triggered by the pandemic is expected to leave lasting scars through lower investment, an erosion of human capital through lost work and schooling, and fragmentation of global trade and supply linkages (World Bank, 2020). In the short- and medium-term economic challenges, the pandemic has brought enormous difficulties for governments in terms of the over-demand for medical care and social support from that part of the population that cannot meet its basic needs the pandemic (Frydman & Phelps, 2020).

Within this picture, similar prominent evidence was the 2008 financial crisis that morphed into multi-faceted social, political, and economic challenges worldwide. While the crisis exposed critical and unsustainable in many countries, the crisis also made clear just how inter-dependent and interlinked the global economies are (Carayannis & Rakhmatullin, 2014). As a result, a new paradigm emerged where government, industry, university, and civil participants work together to co-create the future and drive structural changes far beyond the scope of what any organization/person could do alone (Carayannis & Campbell, 2010). The so-called “quadruple helix model” put a stronger focus on cooperation in innovation via the dynamically intertwined processes of co-opetition, co-evolution, and co-specialization within/across sectors, regions, and eco-systems (Carayannis & Campbell, 2010).

Based on the quadruple helix lessons from the 2008 financial recession, the world was currently exposed to the COVID-19 stakeholders’ needs (e.g., the overflow of patients to areas in atypical hospital care, provision of mechanical ventilation in intensive care units, and assignment of extraordinary health personnel) (Hamele et al., 2018; Juckett, 2006). Although governments have used different technologies for public service and communication with their stakeholders like the e-government (Gil-García & Pardo, 2005; Sharma et al., 2018; Susanto et al., 2017), not all countries have disposed of resources to adopt digital technologies (Fathey et al., 2016). However, social distance restrictions have accelerated the co-creation of multiple digital initiatives among multiple agents to satisfy the numerous COVID-19 health and social urgencies (Barnes & Sax, 2020; Bustinza et al., 2019; Mahajan et al., 2020; Meissner & Kergroach, 2021; Ming et al., 2020). Indeed, most of these entrepreneurial and digital initiatives have been developed altruistically among multiple specialized agents (Mostafa et al., 2020). Indeed, we have observed the emergence of a new phenomenon, “digital social entrepreneurial initiatives,” in the intersection between stakeholders’ needs and digital social initiatives that have emerged in collaboration among multiple social, academic, economic, industrial, political, and civil society (also called N-Helix) to respond to the COVID-19 pandemic (Apple, 2020b; Google, 2020).

The accumulated literature has associated social entrepreneurship (SE) with the emergence of entrepreneurial initiatives that seek to solve certain stakeholders’ social problems (Dacin et al., 2010; Driver, 2017; Robb & Gandhi, 2016; Short et al., 2009), a few of them using open innovation (Hamburg, 2017; Tracey & Stott, 2017) in collaboration among actors (Iqbal et al., 2018). In comparison, digital entrepreneurship (DE) represents the emergence of new entrepreneurial initiatives that incorporated digital technologies like artifacts and platforms(Nambisan, 2017). Following these assumptions, digital social entrepreneurship (DSE) represents entrepreneurial initiatives with social purposes developed by incorporating digital technologies into their business model as a result of the interaction of N-Helix agents (Battisti, 2019; Ghatak et al., 2020; Short et al., 2009). This non-documented phenomenon represents the missing link between innovation, technology, and entrepreneurship literature.

Inspired by this gap, this study examines the relationship between N-Helix collaboration and DSE due to the government’s limited capacity to respond to the stakeholders’ needs satisfaction related to exogenous events (e.g., the COVID-19 pandemic). The proposed conceptual model is based on three ongoing academic debates related to (a) the unrepresentativeness of *the stakeholder theory* in entrepreneurship research; (b) the emergence of *digital social entrepreneurship*; and (c) *the collaboration among multiple agents* (N-Helix). To test our proposed conceptual model, we build a dataset based on the information provided by the two most relevant digital platforms (iOS—Apple store and Android—Google play), the websites of the apps’ developers, and official datasets. Our results show that the government’s limited ability to respond to the pandemic and manage its support services through digital means encourages collaboration among N-Helix partnership agents. These collaborations stimulate DSE that has a positive effect on stakeholder satisfaction. N-Helix partnerships create value through innovation to develop DSE.

After this introduction, Sect. 2 proposes the theoretical framework to understand better the N-Helix response to an exogenous crisis like the COVID-19 pandemic. Section 3 includes the methodological design to test our set of hypotheses. Section 4 shows the main findings of our study that are discussed in light of previous studies. Section 5 offers conclusions, limitations and proposes future research opportunities.

## Theoretical Framework

Given the exposed critical COVID-19 stakeholders’ needs, we are merging three ongoing academic debates. First, *the stakeholder theory* enriches our understanding of the emergence of entrepreneurship initiatives and their process (Venkataraman, 2002). However, it is palpable the unrepresentativeness of this theory in entrepreneurship research (Freeman et al., 2010). Second, while *digital entrepreneurship (DE)* has represented an agile response for achieving twenty-first-century opportunities through new business and innovative models (Kraus et al., 2018; Nambisan, 2017), *social entrepreneurship (SE)* has represented an ability to address social problems (Mair & Marti, 2006). Battisti (2019) suggests an alternative framework that considers socially relevant groups in the entrepreneurial innovation and digital process. Following Battisti’s (2019) reasoning, we argue that the COVID-19 pandemic has raised important questions that should be answered at the intersection of digital technologies, societal needs, and entrepreneurial behaviors emerging a new phenomenon: *digital social entrepreneurship (DSE).* Third, collaboration among diverse social and economic agents (*N-Helix)* facilitates the emergence of global knowledge-intensive initiatives (Del Giudice et al., 2017). These environments facilitate integrated collaborations, co-created shared values, cultivated innovation eco-systems, unleashed exponential technologies, and extraordinarily rapid adoptions (Galvao et al., 2019).

### An exogenous event: the COVID-19 pandemic

The modern world has faced three severe exogenous events related to population deaths (Hamele et al., 2018). First, the 1918 influenza pandemic represented a severe event with nearly 50 million deaths worldwide. Second, the 1957–1968 influenzas that killed approximately 2,000,000 people. Third, the 2009 H1N1 virus caused more than 575,000 deaths. During these events, the lessons learned helped strengthen infection and viral disease monitoring systems (Kotalik, 2005). In these events, the population’s perception of uncertainty is higher in societies with high cultural levels of uncertainty avoidance (Hofstede, 2001). In natural disaster scenarios, the government controls and allocates resources to help the affected populations (Frydman & Phelps, 2020). Therefore, governments are responsible for monitoring, containment, and treatment of health emergencies.

Although governments have implemented initiatives to deal with exogenous events (Gil-García & Pardo, 2005), collaboration among public–private agents emerged to support the population’s needs (Demircioglu & Audretsch, 2020; Fathey et al., 2016). Collaborative initiatives have offered a new way of self-managed uncertainty by allowing them to seek information on their own about their specific information needs (ITU, 2020) and alleviates the demand for digital services in the local government. During the COVID-19 pandemic, apps provide many services such as self-diagnosis, news, quarantine control, infection maps, social distancing, regional trade status, and telemedicine, among many others (Banskota et al., 2020). All these services allow people to feel more secure in an uncertain context, while they might have been dispensable, overlooked, or even annoying in other settings (Dehling et al., 2015). The threat of the COVID-19 pandemic affects all sectors of society. Since the virus’s consequences are potentially deadly, individuals carefully evaluate and catalog all resources and services (Pakpour & Griffiths, 2020). Therefore, users are becoming more demanding for pandemic support services (Kotalik, 2005; Quinn et al., 2013). Based on these arguments, we propose the following hypotheses.

#### H1a

An exogenous event (e.g., COVID-19 pandemic) has a negative effect on the government’s ability to respond to the stakeholders’ needs.

#### H1b

An exogenous event (e.g., COVID-19 pandemic) has a positive effect on the emergence of N-Helix collaborations to the stakeholders’ needs.

#### H1c

An exogenous event (e.g., COVID-19 pandemic) has a positive effect on the creation of digital and social initiatives to respond to the stakeholders’ needs.

#### H1d

An exogenous event (e.g., COVID-19 pandemic) has a negative effect on stakeholders’ needs satisfaction.

### A collaborative response to an exogenous event: The N-Helix model

Previous studies have shown N-Helix models’ evolution by collaborating with governments, universities, industries, and society to respond to specific needs (Fischer et al., 2018, 2019; Kobarg et al., 2018; Lew et al., 2018). In this vein, we have observed dual-helix partnerships (university-industry promoted by the public incentives), triple-helix partnership (university-industry-government), and the quadruple-helix partnership (university-industry-government-society) (Baier-Fuentes et al., 2021; Demircioglu & Audretsch, 2019; Dooley & Kirk, 2007; McAdam & Debackere, 2018). Individually, the government prioritizes sustained structural changes; the university focuses on the generation and transference of knowledge; the industry looks for developing competitive advantages; and social groups look for supporting vulnerable populations (Bărbulescu & Constantin, 2019; Guerrero & Urbano, 2020).

As part of an N-Helix model, multiple actors collaborate to generate/transfer knowledge and innovations into society (Carayannis, E. G., Campbell, 2010; Del Giudice et al., 2017). The knowledge transfer between N-Helix collaborations and exogenous events has positively impacted university-supported firms’ innovation intensity during the 2008 financial crisis (Carboni & Medda, 2021). Indeed, digital technologies facilitate the generation/transference of knowledge and the introduction of new business models or operational processes (He, 2019; Sahut et al., 2019). Knowledge transfer benefits from remote communication technologies, as it has been shown that physical distance between N-Helix collaborators is an essential element to consider for knowledge transfer and innovation (Mukherji & Silberman, 2021). During the COVID-19 pandemic, the insufficient resources/investments in health infrastructures to respond to the population’s needs have motivated the emergence of apps created by collaborating partnerships (Friedman et al., 2020). Therefore, N-Helix collaboration can alleviate the government’s overburden by supporting services related to social distancing, emotional support, remote medical services, monitoring of active cases, among other tools that can be implemented through information and communication technologies (Banskota et al., 2020). Based on these arguments, we propose the following hypotheses.

#### H2

In the context of an exogenous event (e.g., COVID-19 pandemic), the reduction in the government’s response capacity is positively related to the creation of H-Helix collaboration.

### The stakeholders’ needs satisfaction: N-Helix via digital social entrepreneurship (DSE)

Previous studies have recognized the positive effect of N-Helix collaborations (e.g., industry, government, university, non-profit organizations, civil society) on multiple stakeholders’ needs satisfaction (Carayannis & Campbell, 2010; Fischer et al., 2020; Blair-Fuentes et al., 2021). Nevertheless, the literature has also recognized many challenges in the configuration of N-Helix collaborations, such as response-times, bureaucracy, exclusion feelings, ethical dilemma, and intellectual property (Brannback et al., 2008; Goyal et al., 2016; Kraus et al., 2018).

Although N-Helix collaborations imply multiple challenges (Iqbal et al., 2018; Saiz-Álvarez & Palma-Ruiz, 2019), social distance restrictions and the COVID-19 stakeholders’ needs have encouraged N-Helix agents to look for a crucial intersection between social entrepreneurship (SE) and digital entrepreneurship (DE) initiatives that have configurated the called digital social entrepreneurship (DSE). In this new scenario, digital technologies have been an invaluable resource for multiple N-Helix collaborations oriented to create an agile response to the global pandemic effects (Ghatak et al., 2020; Short et al., 2009; Siegel & Guerrero, 2021). DSE can come in various sizes, depending on the resources involved in its creation. In general, those large-scale digital social initiatives are developed collaboratively between two or more N-Helix agents. The N-Helix model made a stronger focus on cooperation in innovation, the dynamically intertwined processes of co-opetition, co-evolution, and co-specialization within/across sectors, regions, and eco-systems (Carayannis & Campbell, 2010; Kolesnikov et al., 2019). We assume that exogenous events as the COVID-19 pandemic have intensified DSE development by N-Helix actors (e.g., government, research centers, universities, industries, social organizations, and society). This type of collaboration allowed sharing available resources and specialized knowledge to satisfy societal needs with reduced costs, improved quality, and global coverage (Minshall et al., 2008; United Nations, 2020b). This sense highlights the importance of networks and institutional spaces for transferring technology and innovation to promote DSE towards a high-value proposition (Kruger & Steyn, 2020; Panetti et al., 2020). Indeed, N-Helix agents have also faced significant efforts/tensions to develop these DSE with a large-scale impact, and in some cases, the DSE has generated the non-satisfaction of the COVID-19 stakeholders’ satisfaction.

According to Schumpeter (1976: 83–84), every business strategy acquires true significance only against that process’s background and within the situation created by it. This reflection explains why management literature has considered stakeholders’ crucial role in defining business strategies (Freeman et al., 2010). Likewise, according to Venkataraman (2002), the stakeholder theory needs to consider the entrepreneurial process as a legitimate method of bringing about stakeholder equilibration; as well as, the entrepreneurship theory must consider stakeholder equilibration explicitly (Venkataraman, 2002). Otherwise, both theories are incomplete. In this vein, entrepreneurship should help to identify and exploit opportunities to improve the well-being of individuals, groups, and communities through solutions driven by digital technologies (Goss, 2005; Ratten, 2018). In this context, the entrepreneur acts as a catalyst between the opportunities that emerge from an exogenous event and the knowledge spillover that allows the introduction of innovations and new markets (Caiazza et al., 2020). We assume that DSE had emerged to address the N-Helix stakeholders’ needs on a local, national, and global scale (Battisti, 2019). Therefore, DSE could be the driver of sustainable innovation of N-Helix collaboration (Hinings et al., 2018). Based on these arguments, we propose the following hypotheses.

#### H3a

In the context of an exogenous event (e.g., COVID-19 pandemic), N-Helix is positively related to the creation of DSE.

#### H3b

In the context of an exogenous event (e.g., COVID-19 pandemic), DSE is positively related to stakeholders’ needs satisfaction.

#### H3c

In the context of an exogenous event (e.g., COVID-19 pandemic), DSE positively mediates the relationship between N-Helix and stakeholders’ needs satisfaction.

### Proposed conceptual model

**Fig. 1:**
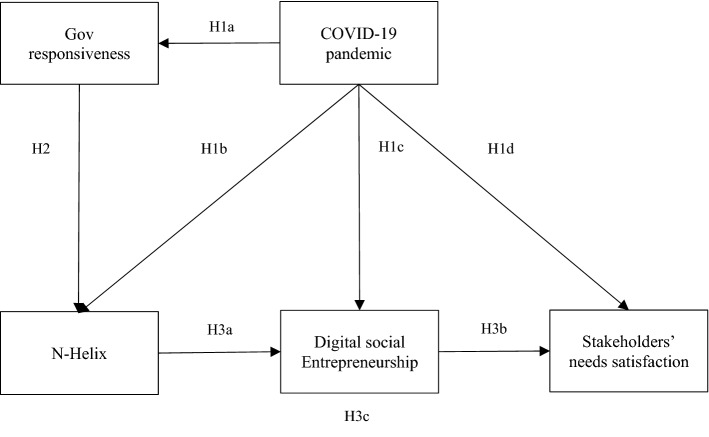
Conceptual Model. *Source:* Authors

Based on the literature review, Fig. 1 shows our proposed conceptual model.

Our conceptual model proposes that an exogenous event (e.g., the COVID-19 pandemic) has a negative influence on the government’s ability to respond to social demands (Miao et al., 2021) (H1a). The prioritization of government health spending in pandemic times means that non-emergency expenditures will be postponed (Prasad Das & Nundy, 2020). In unstable economies, resource shortages will be even more damaging, even to the extent that they will not meet the health care needs of their citizens during the pandemic (Juckett, 2006). In this view, the COVID-19 pandemic will positively impact N-Helix collaboration and DSE (H1b). An exogenous event represents a concern for all sectors of society. N-Helix stakeholders can be motivated to support the government and citizens in addressing the health crisis through digital services and media such as mobile applications (Marceau, 2002). Given the global contingency, N-Helix collaborations offer their services to citizens free of charge (Apple, 2020b; Google, 2020). The purpose is social well-being, which shows the pandemic’s positive influence on DSE (Berger et al., 2004) (H1c).

We propose that the COVID-19 pandemic will also have a negative effect on the stakeholders’ assessment of DSE (H1d). The COVID-19 consequences are related to individuals’ concerns about the reputation of sources that disseminate pandemic-related information and services (Pakpour & Griffiths, 2020). This fact has increased the attention to reliability sources due to the pandemic’s seriousness has led to more demanding assessments by stakeholders.

We also suggest a negative relationship between insufficient government capacity to satisfy stakeholder needs through digital media and N-Helix collaborations (H2). The government’s response capacity reduction increases the partnership between N-Helix actors (Battisti, 2019). Therefore, we expect greater collaboration among N-Helix agents stimulates DSE (H3a).

The DSE’s altruistic nature allows users to access free services that they could not obtain in other ways (Chesbrough & Di Minin, 2014). Individuals will better value service if they do not have to pay for it; in this sense, DSE will positively affect stakeholder satisfaction (H3b). In this vein, DSE will increase users’ satisfaction since N-Helix initiatives can provide better quality resources and disseminate high-level innovation to stakeholders (Bonaccorsi & Piccaluga, 1994; Galvao et al., 2019). Therefore, we anticipate a positive effect of the N-Helix collaboration on stakeholder satisfaction through DSE (H3c).

## Methodology

### Data collection

Dynamic research developed during two doctoral elective courses (social entrepreneurship and digital entrepreneurship) from March 2020 to August 2020. We build a dataset using the two most relevant digital platforms: iOS (Apple store) and Android (Google play) (Srinivasan & Venkatraman, 2018). Specifically, we paid attention to the apps created from January 2020 to July 2020 worldwide to respond to the COVID-19 pandemic. We obtained a sample of 130 mobile validated applications from 48 countries. The applications included in the sample were selected considering the support of a government, university, foundation, or company. It allowed us to analyze the origin and content of the apps with information from additional reliable sources. We complement our dataset by researching websites of developers (universities, governments, industrial actors, and non-profit organizations) and secondary datasets about COVID-19 statistics at the country level (i.e., United Nations, World Health Organization, International Monetary Fund). From January to July 2020, Fig. 2 shows the number of COVID-19 infections globally and the created apps to reduce the COVID-19 pandemic’ effects (Apple, 2020b; ECDC, 2020; Google, 2020).

**Fig. 2:**
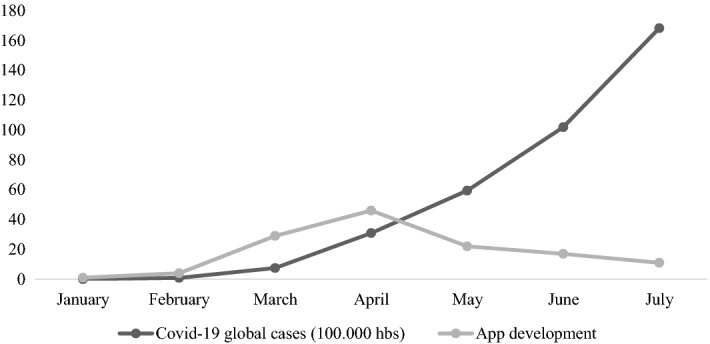
The Covid-19 pandemic world progress versus the total number of apps related to. Source: European Centre for Disease Prevention and Control, Google Play and App Store

From January to date, the leading society’s need has been reliable information on the pandemic’s progress (UNESCO, 2020). The reputation of institutions has been insufficient to trust their initiatives’ credibility (Liao et al., 2020; Quinn et al., 2013). According to UNITAR (2020), inter-institutional collaborations have built a more reliable reputation than alone. The COVID-19 pandemic has required various specialized resources (e.g., computer support, academic research, medical equipment, medical assistance, diagnosis and treatment, communication) (WHO, 2020a). It is unlikely that a single organization will have all these resources, and it is costly to acquire them from other specialized actors (Apple, 2020a).

### Variables

*Our dependent variable is the stakeholders’ needs satisfaction* (Melin et al., 2020). This variable is measured using a Likert scale (1 lowest to 5 highest) to capture the app’s ranking satisfaction. This information came from digital platforms (iOS and Android) and provided information about how much each app was beneficial for its users.

*Regarding independent variables,* we used a set of independent variables. First, *the COVID-19 pandemic* represents the level of danger of the virus for each country (Hamele et al., 2018). This variable was measured using the number of deaths per 100,000 habitants and the number of doctors and hospital beds per 10,000 habitants. The information came from the 2019 Human Development Report 2019 (UNDP, 2019) and the European Centre for Disease Prevention and Control (ECDC, 2020). Second, *government responsiveness* is a binary variable that takes the value one if it is developed only by the government and otherwise zero (Guerrero, et al., 2020a; Guerrero, et al., 2020b; Susanto et al., 2017). Third, the variable *digital social entrepreneurship* is a binary variable that captures value one when the app was created for social purposes (satisfy the COVID-19 needs) and zero when the app was created for commercial purposes (Battisti, 2019; Ghatak et al., 2020; Luke & Chu, 2013; Short et al., 2009). Fourth, the *N-Helix* is a count variable that indicates how many stakeholders participate in developing the app for social purposes (e.g., government, university, industry, non-profit organizations) (Baier-Fuentes et al., 2021; Guerrero, et al., 2020a; Guerrero, et al., 2020b; Lazzarotti & Manzini, 2009; Santos & Mendonça, 2017).

*Regarding control variables,* we used several controls at the country level. Table 1 shows the distribution of the apps in the sample by their country of origin. Table 2 shows the descriptive statistics of the sample, and Table 3 shows the correlation matrix.

**Table 1:** App Distribution by Country. Source: Google Play and App Store

# Country	Total App	Government App	Digital social app	N-Helix App
Germany	2	0	2	2
Saudi Arabia	3	2	1	1
Argentina	1	1	0	0
Armenia	1	1	0	0
Australia	2	0	1	0
Austria	1	0	1	1
Bolivia	1	1	0	0
Brazil	1	1	0	0
Canada	6	3	3	2
Chile	4	1	2	0
Colombia	2	1	1	1
Dubai	1	1	0	0
United Arab Emirates	1	1	0	0
Scotland	1	1	0	0
Spain	9	1	8	3
Estonia	1	0	1	0
France	1	0	1	1
Ghana	1	1	0	0
Global	1	0	1	0
Netherlands	3	0	3	0
India	6	2	4	1
Indonesia	1	0	1	1
Ireland	3	1	2	0
Israel	1	0	1	0
Italy	1	1	0	0
Jamaica	1	1	0	0
Jordan	1	0	1	1
Luxemburg	1	0	1	0
Mali	1	1	0	0
Mexico	4	3	1	0
Nepal	1	0	1	1
Pakistan	1	1	0	0
Peru	3	1	2	0
Poland	1	1	0	0
Qatar	1	0	1	1
United Kingdom	6	0	5	4
Czech Republic	1	0	0	0
Romania	2	0	1	0
Russia	3	1	2	0
Switzerland	2	1	1	1
Thailand	1	0	1	1
Turkey	1	0	1	0
USA	38	5	28	18
Ukraine	1	0	1	1
Uruguay	1	1	0	0
Vietnam	2	0	2	2
South Korea	1	0	1	1
Japan	1	1	0	0

**Table 2:** Descriptive statistics

Variables	Measures	Source	Mean	S.D
COVID-19 pandemic	Number of hospital beds per 10,000 habitants	Human development report	37.00	25.00
	Number of doctors per 10,000 habitants	Human development report	25.68	10.53
	Number of deaths per 100.000 habitants	European Centre for Disease Prevention and Control	32.05	20.88
Gov responsiveness	A binary variable takes the value one if the app is developed only by the government and zero otherwise	Google Play and App Store	0.31	0.46
N-Helix	Count variable that indicates how many stakeholders participate in the development of the app for social purposes	Google Play and App Store	1.40	0.69
Digital social entrepreneurship	A binary variable that captures value one when the app was created for social purposes and zero when it was created for commercial purposes	Google Play and App Store	0.65	0.48
Stakeholders’ needs satisfaction	A Likert scale (1 lowest to 5 highest) to captures the users’ ranking satisfaction per app	Google Play and App Store	3.11	1.72

**Table 3:** Correlation matrix

	COVID-19 pandemic	Gov responsiveness	N-Helix	Digital social entrepreneurship	Stakeholders’ needs satisfaction
COVID-19 pandemic	1.000				
Gov responsiveness	− 0.240**	1.000			
N-Helix	0.122	− 0.389***	1.000		
Digital social entrepreneurship	0.138	− 0.325***	0.569***	1.000	
Stakeholders’ needs satisfaction	− 0.180	0.192***	0.125	0.149*	1.000

*/**/*** Significance level 0.1/0.05/0.01

### Method

We use Partial Least Squares Structural Equation Modeling (PLS-SEM) using SmartPLS software (Ringle et al., 2015). Our sample is suitable for the implementation of this technique (Wong, 2013). This method is ideal for testing multiple relationships between variables and estimating the direct and indirect effects (Matthews et al., 2018).

## Results and Discussion

**Fig. 3:**
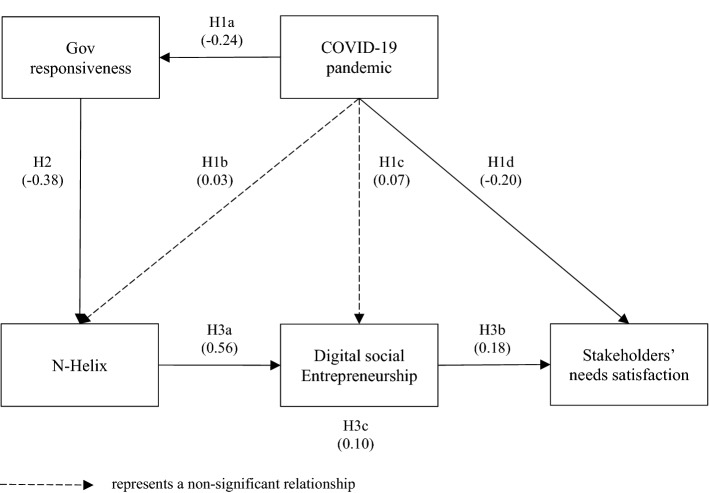
Hypothesis testing. *Source* Authors

Tables 4 and 5 show the direct and indirect effects, respectively. Indeed, Fig. 3 shows the testing of the proposed hypotheses.

**Table 4:** PLS-SEM model results

Measures		Mean	S.D
*Path Coefficients*			
COVID-19 pandemic → Gov responsiveness	–	0.24**	0.11
COVID-19 pandemic → N-Helix		0.03	0.10
COVID-19 pandemic → Digital social entrepreneurship		0.07	0.09
COVID-19 pandemic → Stakeholders’ needs satisfaction	–	0.20*	0.12
Gov responsiveness → N-Helix	–	0.38***	0.05
N-Helix → Digital social entrepreneurship		0.56***	0.10
Digital social entrepreneurship → Stakeholders’ needs satisfaction		0.18**	0.09
*R-Square*			
Gov responsiveness		0.06	0.05
N-Helix		0.15***	0.03
Digital social entrepreneurship		0.33***	0.10
Stakeholders’ needs satisfaction		0.06	0.05
*Total Effects*			
COVID-19 pandemic → Gov responsiveness	–	0.24**	0.11
COVID-19 pandemic → N-Helix		0.12	0.10
COVID-19 pandemic → Digital social entrepreneurship		0.14	0.09
COVID-19 pandemic → Stakeholders’ needs satisfaction	–	0.18	0.12
Gov responsiveness → N-Helix	–	0.38***	0.05
Gov responsiveness → Digital social entrepreneurship	–	0.21***	0.05
Gov responsiveness → Stakeholders’ needs satisfaction	–	0.04*	0.02
N-Helix → Digital social entrepreneurship		0.56***	0.10
N-Helix → Stakeholders’ needs satisfaction		0.10*	0.05
Digital social entrepreneurship → Stakeholders’ needs satisfaction		0.18**	0.09

*/**/*** Significance level 0.1/0.05/0.01

**Table 5:** PLS-SEM model results–indirect effects

Measures		Mean	S.D
*Specific Indirect Effects*			
COVID-19 pandemic → Gov responsiveness → N-Helix		0.09**	0.04
COVID-19 pandemic → Gov responsiveness → N-Helix → Digital social entrepreneurship		0.05**	0.03
COVID-19 pandemic → N-Helix → Digital social entrepreneurship		0.02	0.06
COVID-19 pandemic → Digital social entrepreneurship → Stakeholders’ needs satisfaction		0.01	0.02
COVID-19 pandemic → Gov responsiveness → N-Helix → Digital social entrepreneurship → Stakeholders’ needs satisfaction		0.01	0.01
COVID-19 pandemic → N-Helix → Digital social entrepreneurship → Stakeholders’ needs satisfaction		0.00	0.01
Gov responsiveness → N-Helix → Digital social entrepreneurship	–	0.21***	0.05
Gov responsiveness → N-Helix → Digital social entrepreneurship → Stakeholders’ needs satisfaction	–	0.04*	0.02
N-Helix → Digital social entrepreneurship → Stakeholders’ needs satisfaction		0.10*	0.05
*Total Indirect Effects*			
COVID-19 pandemic → N-Helix		0.09**	0.04
COVID-19 pandemic → Digital social entrepreneurship		0.07	0.06
COVID-19 pandemic → Stakeholders’ needs satisfaction		0.02	0.02
Gov responsiveness → Digital social entrepreneurship	–	0.21***	0.05
Gov responsiveness → Stakeholders’ needs satisfaction	–	0.04*	0.02
N-Helix → Stakeholders’ needs satisfaction		0.10*	0.05

*/**/*** Significance level 0.1/0.05/0.01

### The effect of the COVID-19 pandemic

The COVID-19 pandemic has a negative impact on local government response capacity (β_1a_ = -0.240; *p*-value < 0.05), supporting H1a. A plausible explanation is that extraordinary demands on public administration during the pandemic require that governments prioritize citizens’ needs according to a criterion of urgency (Leal Filho et al., 2020). Therefore, the government cannot meet all the population’s needs simultaneously. The main explanation is the limited resources, technical capacity, or the overflow in demand for public services (Frydman & Phelps, 2020). Despite there are some economies that respond to the citizens’ needs during an external event, the magnitude of infections during a pandemic overlaps the countries’ capacities across the globe (Bohlken et al., 2020). Several countries prepared for a global viral/infectious outbreak because of the 2009 H1N1 pandemic (Kotalik, 2005). However, the rapid spreading of the COVID-19 pandemic and many deaths worldwide show the greater danger of this disease concerning other health crises (Krist et al., 2020). Indeed, the COVID-19 pandemic also has a negative effect on stakeholders’ needs satisfaction (β_1d_ =  − 0.204; *p*-value < 0.10), supporting H1d. The main citizens’ concern is related to the lethality and the authorities’ recommendations (Quinn et al., 2013; UNESCO, 2020). Given the nature of the COVID-19 pandemic, it has increased users’ preference for mobile applications developed by institutions with a good reputation (Mahajan et al., 2020). As a result, the population demands the assessment of apps related to the COVID-19 pandemic. We do not find strong evidence to support the effect of the COVID-19 pandemic on N-Helix (H1b) and DSE (H1c). The model shows a significant indirect effect related to the intensity of the COVID-19 pandemic and the involvement of N-Helix in the development of DSE (see Table 5). During an exogenous event, government authorities control decisions and resources related to the exogenous event (Mahajan et al., 2020). In this COVID-19 pandemic, governments and global organizations have called for respecting the restrictions imposed and following only information and communication from official sources (UNESCO, 2020). Given these restrictions, the government controls but also stimulates the collaboration of public and private organizations.

### N-Helix configuration in times of the COVID-19 pandemic

Our results show a negative relationship between government responsiveness and the N-Helix collaboration (β_2_ = – 0.381; *p*-value < 0.01), supporting H2. The plausible explanation is that a decrease in the government’s response capacity to exogenous events (e.g., the COVID-19 pandemic) generates an increase in the collaboration of multiple actors (e.g., universities, research organizations, industries, social organizations). In this scenario, N-Helix agents will detect the governments’ limitations and the population’s needs. Based on this information, N-Helix agents will collaborate to develop initiatives to satisfy the population’s needs and transfer knowledge to respond to them. In the COVID-19 pandemic case, N-Helix is looking to use digital resources to help track, monitor, and follow-up infected individuals and provide multiple types of supports (Apple, 2020a). The collaboration among N-Helix agents helps fill societal needs and bring high-level innovations within a global solidarity scenario oriented to address the COVID-19 pandemic (Banskota et al., 2020; Mahajan et al., 2020; WHO, 2020a).

### Digital social entrepreneurship as a response to the COVID-19 pandemic

Our results show that N-Helix is positively related to creating DSE (β_3a_ = 0.560; *p*-value < 0.01), supporting H3a. The analyzed N-Helix collaborations are related to developing, updating, and maintaining digital applications related to the COVID-19 pandemic. As DSE represents a way to respond to the COVID-19 pandemic, most digital applications have been developed using private–public donations (WHO, 2020a). This altruistic funding system allows apps to be accessed and used free of charge by multiple users regardless of their economic situation or geographic location. The N-Helix partnerships are primarily motivated by the social purpose of supporting multiple users during the COVID-19 pandemic. Regarding the users’ needs satisfaction, the results show the positive effect of DSE on stakeholders’ needs satisfaction (β_3b_ = 0.177; *p*-value < 0.05), supporting H3b. These results show some insights into the benefits captured by the apps’ users during the COVID-19 pandemic. A plausible explanation could be that the users have access to multiple solutions to their needs that they could not obtain by other means during the pandemic times (Chesbrough & Di Minin, 2014). Therefore, in times of pandemic, digital entrepreneurial initiatives’ social orientation represents the mechanism to access free supports to satisfy their stakeholders’ needs (Liao et al., 2020). Table 5 also shows a positive indirect effect between the N-Helix and the stakeholders’ needs satisfaction through DSE (β_3c_ = 0.099; p-value < 0.10), supporting H3c. As a result of the sharing of resources, capabilities, reputations, and risks among multiple agents (universities, businesses, non-governmental organizations, and government), the N-Helix developers legitimized DSE. Therefore, DSE is the vehicle through N-Helix agents that contribute to users’ confidence and needs satisfaction. In this view, the N-Helix DSE added value through joint innovative and entrepreneurial initiatives to improve multiple stakeholders’ satisfaction (Cunningham et al., 2018). Therefore, we assume a mediation effect of DSE in the relation between N-Helix and stakeholders’ satisfaction.

## Conclusion

This study examined the relationship between N-Helix collaboration and DSE due to the government’s limited capacity to respond to the stakeholders’ needs satisfaction related to exogenous events (e.g., economic crises, natural disasters, pandemics). We propose a conceptual framework that was tested with 130 apps related to the COVID-19 pandemic provided by Google Play and App Store from March 2020 to July 2020. Our model shows adequate measures of fit and supports our hypotheses.

An exogenous event like the COVID-19 pandemic reduces the government’s ability to respond and, in turn, produces a decrease in stakeholder satisfaction. A decline in agile government responsiveness stimulates the formation of N-Helix collaborations that act as support entities during exogenous events to serve stakeholders who have fallen beyond the state’s reach. Since a good part of the N-Helix initiatives pursues altruistic purposes for transferring technology, knowledge, and innovation to the most vulnerable or neglected sectors, we found a positive relationship between N-Helix initiatives’ formation and DSE. Also, DSE positively influences stakeholder satisfaction and acts as a mediator between N-Helix initiatives and higher stakeholder satisfaction. Our results show that N-Helix initiatives, mainly DSE, function as catalysts of knowledge transfer and innovation for stakeholder satisfaction when the government cannot meet the population’s needs. In this sense, exogenous global scope events, such as the COVID-19 pandemic, stimulate the emergence of social initiatives intensive in innovation and high-value creation through the need for support from governmental institutions to face these situations.

### Implications for academics

Based on our results, we contribute to three ongoing academic debates. *The first academic debate is related to the stakeholder theory.* Our results show that N-Helix collaborations have emerged as an agile response to meet stakeholder needs in the context of an exogenous event, supporting the government in areas it cannot achieve (Demircioglu & Audretsch, 2020; Frydman & Phelps, 2020). During exogenous events, users demand more support services, testing and pressuring the capacity of government, industries, universities, and civil society, and the entire entrepreneurial eco-system in general (Miao et al., 2021; Prasad Das & Nundy, 2020; Quinn et al., 2013). N-Helix collaborations through DSE provided innovation/technology-based solutions to improve users’ quality of life during the coronavirus restrictions (Del Giudice et al., 2017; Goss, 2005; Ratten, 2018). This study has evidenced the close relationship between N-Helix collaborations, DSE, and stakeholder satisfaction. Like Caiazza et al. (2020), the DSE represents a catalyst between opportunities that arise during an exogenous event and knowledge/technology transfer. In this assumption, N-Helix collaborations generate social/health impacts, promote agile DSE solutions, and increase stakeholder satisfaction. The study shows that the phenomenon of DSE created under the umbrella of N-Helix collaborations opens a new window of research opportunities to enrich the stakeholder theory by including a contingent approach (e.g., the analysis of exogenous events like a crisis, pandemics, natural disasters), as well as the generation/spread of accessible open innovations at large scale.

*The second academic debate is related to digital entrepreneurship and social entrepreneurship literature.* The intersection between digital technologies, societal needs, and entrepreneurship opens up a new edge in analyzing how society can address the challenges of exogenous events like the COVID-19 pandemic (Battisti, 2019). N-Helix collaborations through DSE have endorsed a solidarity-technology-based economy (Iqbal et al., 2018; Saiz-Álvarez & Palma-Ruiz, 2019). Indeed, our results show how the coincidence between SE, DE, and N-Helix collaborations has globally supported health, social, economic, and emotional needs (Minshall et al., 2008; United Nations, 2020a, 2020b). This study contributes to entrepreneurship, innovation, and management literature by offering the DSE new approach that could reduce the number of vulnerable groups and generate large-scale implications for solving social needs worldwide during exogenous events (e.g., the COVID-19 pandemic). Related to open innovation literature (Chesbrough & Di Minin, 2014), DSE also represents a vehicle to generate accessible innovations for social purposes, as well as a better understanding of how global solidarity enables the spread of knowledge, technologies, and digital solutions to deal global exogenous events (e.g., the COVID-19 pandemic). Indeed, future research should study in-depth the multiple efforts, challenges, and tensions associated with the development of these DSEs through N-Helix agents (Siegel & Guerrero, 2021).

*The third academic debate is related to N-Helix collaborations*, results show that the government’s inability to address the social needs arising from an exogenous event such as the COVID-19 pandemic motivates the configuration of N-Helix collaborations among multiple agents (Battisti, 2019; Marceau, 2002). N-Helix collaborations facilitate the creation of knowledge/technology-intensive initiatives and benefit from innovation eco-systems to create social impact through DSE (Del Giudice et al., 2017; Galvao et al., 2019). N-Helix collaborations faced multiple tensions and challenges to successfully integrate DSE in the adverse conditions arising from the COVID-19 scenario. Concretely, results show that the COVID-19 pandemic influences the N-Helix DSE’s creation and the agile government responsiveness. N-Helix collaborations occur spontaneously in uncertain conditions triggered by the quality of coverage/response to the government’s actions (Friedman et al., 2020). This study contributes by exposing the emergence of DSE due to N-Helix collaboration and extending the notion of the quadruple helix approach (Carayannis & Rakhmatullin, 2014). The intersection between N-Helix collaborations and DSE has a significant positive impact on achieving various stakeholders’ satisfaction. The explanation is that DSE provides open knowledge-intensive solutions to complex social problems. From this perspective, stakeholders can benefit from N-Helix cooperation by accessing innovative, entrepreneurial, and digital solutions. Given the N-Helix agents’ capabilities, DSE is an efficient alternative where the government has limited resources/capabilities to overcome exogenous events.

### Implications for N-Helix agents and stakeholders

Two implications emerge from our results. *For N-Helix agents,* we provide insights into the global impact of DSE (apps) developed by N-Helix actors to satisfy the stakeholders’ COVID-19 pandemic needs. It is a relevant demonstration and legitimization about how the N-Helix collaborations could redefine economic and social agendas post-pandemic. For instance, during the 2008 financial recession, European policy-makers decided to foster quadruple helix collaborations. In this vein, post-COVID-19 pandemic, policy-makers may foster DSE as an alternative to face the challenges in the new socio-economic configuration. N-Helix and DSE have positively satisfied the stakeholders’ needs, implies that this type of initiative shows good performance and is desirable for society, considering the context of an exogenous event. Based on the 2018 financial crisis lessons, governments may learn to increase their capacity to respond to crises by engaging N-Helix collaborations, which could alleviate the state’s burden and make better use of public resources. *For stakeholders*, we provide insights into the societal benefits of digital, innovative, and entrepreneurial initiatives developed by N-Helix actors. It reinforces and raises the many agents’ participation in actions to support societal and economic recovery post-COVID-19 pandemic or another exogenous event (e.g., natural disasters). N-Helix stakeholders can benefit from knowledge/technology transfer by accessing better resources to generate a more significant impact in their digital social initiatives (Carayannis & Campbell, 2010; Kolesnikov et al., 2019). DSE could globally help vulnerable groups by improving the well-being and generating high-value social impacts through collaboration among multiple actors.

### Limitations and research agenda

Our study has some limitations that represent opportunities for future research. *The first limitation* is related to the size of our sample. We are studying an exogenous phenomenon in real-time (e.g., the COVID-19 pandemic). Therefore, our sample was conditioned to the number of applications created during our period of analysis. A natural extension of this study is collecting data during the months associated with the COVID-19 pandemic. *The second limitation* is related to stakeholders’ satisfaction measurement. The users’ rating is voluntary. It explains why the total number of downloads is not the same as the number of users’ rates. A natural extension demands new objective/subjective metrics that capture users’ satisfaction and feedback. *The third limitation* is related to environmental conditions. Although country-level conditions control us, the country level demands in-depth analysis. By increasing the sample size, we hope to extend this analysis.

Future research could adopt a multidisciplinary theoretical approach (e.g., focusing on N-Helix social initiatives’ value creation, as Guerrero, et al., 2020b and Siegel & Guerrero, 2021 suggested). *First, strategic management literature offers* interesting conceptual approaches like resource-based view, knowledge management, and complexity approaches to understanding how, in challenging times, N-Helix collaborations are strategically configured in terms of share resources/capabilities, define the strategies agilely, manage conflicts/tensions agilely, and manage intellectual outcomes that are generated via DSEs. *Second, economic and political science literature offers* interesting conceptual approaches in terms of socio-economic responses/impacts of exogenous events and how the government strategically manages internal crises generated by external crises. *Third, entrepreneurship literature offers* multiple lenses to understand motivations, decision-making processes among entrepreneurs and N-Helix agents (including the convergence between entrepreneurial and innovation eco-system agents), the DSE’s evolutive trajectory, and configuration of a new digital-social-entrepreneurial identify. *Third, innovation literature offers* interesting approaches to understanding technology and knowledge processes among N-Helix collaboration in challenging times, such as intellectual protection, knowledge transfer, and knowledge commercialization (Hayter & Link, 2021; Siegel & Guerrero, 2021).

Future research represents an opportunity to continue discussing the DSE challenges, what they are, how stakeholders face them, and how these can be implemented in a post-COVID-19 scenario. This research also suggests a provocative discussion regarding the potential dual impacts (positive and negative) of DSE in society. We hope to encourage academic debate regarding new conceptual/empirical trends in DSE and N-Helix collaborations’ contributions in uncertain times.
